# Effects of resistant starch interventions on circulating inflammatory biomarkers: a systematic review and meta-analysis of randomized controlled trials

**DOI:** 10.1186/s12937-020-00548-6

**Published:** 2020-04-15

**Authors:** Mahsa Vahdat, Seyed Ahmad Hosseini, Golsa Khalatbari Mohseni, Javad Heshmati, Mehran Rahimlou

**Affiliations:** 1 Nutrition and Metabolic Disease Research Center, Ahvaz Jundishapur University of Medical Sciences, Ahvaz, Iran; 2 Nutrition Department, Faculty of Paramedicine, Ahvaz Jundishapur University of Medical Sciences, Ahvaz, Iran; 3 Department of Nutritional Science, School of Nutritional Science and Food Technology, Kermanshah University of Medical Sciences, Kermanshah, Iran

**Keywords:** Inflammation, Resistant starch, CRP, TNF-α, IL-6, Meta-analysis

## Abstract

**Purpose:**

This study aimed to summarize earlier studies on the effects of RS consumption on the serum levels of inflammatory biomarkers.

**Methods:**

A comprehensive search was done in the electronic databases that published from 1988 up to May 2019. Two reviewers independently performed screening, data extraction, and risk-of-bias assessment. We used from the effect size, as estimated by the mean difference to perform the fixed method meta-analysis.

**Results:**

Overall, 13 studies with 14 effect sizes met the inclusion criteria and were included in the final analysis. Sample size of these studies ranged from 15 to 75 and intervention duration ranged from 4 to 14 weeks. Meta-analysis revealed that higher consumption of resistant starch caused a significant reduction in the interleukin 6 (weighted mean difference = − 1.11 pg/mL; 95% CI: − 1.72, − 0.5 pg/mL; P = < 0.001) and tumor necrosis factor alpha (weighted mean difference = − 2.19 pg/mL; 95% CI: − 3.49, − 0.9 pg/mL; *P* = 0.001) levels. However, no significant changes were found in C-reactive protein concentration (weighted mean difference = − 0.21 mg/L; 95% CI: − 1.06, 0.63 mg/L; *P* = 0.61). Moreover, the changes in interleukin 6 concentration was dependent on study quality and intervention duration.

**Conclusion:**

The current meta-analysis indicated that RS intake can improve some inflammatory biomarkers. More research, with a large sample sizes and accurate design is recommended.

## Introduction

Low grade systematic inflammation is involved in the development and progression of several metabolic conditions [1, 2]. Inflammation is a protective mechanism that is vital to health [3], but a chronic, systemic inflammation was observed in many diseases, such as cardiovascular disease, obesity, metabolic syndrome, non-alcoholic fatty liver and type 2 diabetes [4, 5]. Therefore, inflammatory markers are known as a risk factor for disease prediction [6]. Inflammatory biomarkers such as cytokines, chemokines, adhesion molecules and acute-phase reactants play an important role in the chronic disease pathogenesis [7].

According to previous studies, diet can play a role in reducing inflammation [8, 9]. For example, a high-fiber diet can help to reduce inflammatory cytokines by increasing the production of short chain fatty acids in the colon [10]. Recently, the role of microbial flora in inflammation and metabolic disorders has attracted much attention [11].

Resistant starch (RS) is a part of starch in the diet that is not digested and absorbed in the small intestine and is fermented in the colon to produce short chain fatty acids (SCFA) [12]. It is considered as a type of dietary fiber. Resistance of starch is affected by the amylopectin and amylose ratio. Amylose tends to be digested more slowly and to a lesser extent than amylopectin [13]. Resistant starch is divided into five different types based on the origin and physical properties of starch [13]. It can produce more butyrate in comparison to other prebiotics. Butyrate is the main SCFA that is produced from the fermentation of RS and acts as an anti-inflammatory agent [14–16].

Studies on the effect of resistant starch on inflammatory markers are conflicting. Some studies showed a significant decrease in inflammatory markers after RS consumption compared to placebo [17, 18], but the results of some other studies were inconsistent [19, 20].

Although studies have been conducted on the effect of resistant starch on inflammatory markers, as far as we know there is no study that summarizes the findings of previous studies. Therefore, the purpose of this study was to provide a systematic review and meta-analysis of extant literature about the effect of RS consumption on several inflammatory biomarkers.

## Method

### Study identification and selection

The reporting of this review is aligned with the Preferred Reporting Items for Systematic Reviews and Meta-Analyses (PRISMA) [21]. Systematic search was done from 1988 up to 20 May, 2019 in electronic databases (PubMed, clinicaltrials.gov, Scopus, EMBASE, Cochrane Library and World Health Organization International Clinical Trial Registry Platform). Also, the reference list of related articles was hand searched for additional relevant studies. Combinations of the terms “Resistant starch” OR “resistant maltodextrin” OR “resistant dextrin” OR “indigestible dextrin” OR OR “indigestible starch” OR “high amylose starch” OR “slowly digestible starch” AND inflammation OR inflammatory OR “inflammatory factors” OR “C reactive protein” OR “C-reactive protein”OR “high-sensitivity C-reactive protein” OR hs-CRP OR interleukin-6 OR “interleukin 6” OR IL-6 OR “tumor necrosis factor-” OR “tumor necrosis factor” OR TNF-α were searched. We included only studies with the randomized method and measurement of ≥1 of the primary outcomes. Other type of the studies (observational studies, nonclinical studies and uncontrolled trials) and studies that resistant starch was used in combination with other dietary components, supplements or drugs were not included in our study.

### Data extraction and quality assessment

Data were extracted independently by the 2 investigators (MR and MV) according to eligibility criteria (Table 1). The percentage agreement in study eligibility and a *κ* statistic were calculated to check concordance between reviewers [22]. In the event of disagreement between the two researchers, SH cross-examined doubtful data, with a decision being made after a consensus meeting. If a study had insufficient information, we would email to the corresponding author and ask him information.

**Table 1:** Inclusion and exclusion criteria following the PICOS approach^a^

PICOS	Inclusion and exclusion criteria	Data extraction
**Participants**	Adult population’s ≥ 18 and ≤ 65 y with or without disease. Studies with a median age between these values were eligible. Participants with mean age ≤ 18 y or nonclinical studies were excluded.	Age, sex, gender, sample size, location, inclusion and exclusion criteria
**Intervention**	Resistant Starch defined as “resistant maltodextrin”, “resistant dextrin”, “indigestible starch”, “high amylose starch” or any other compound defined by the author as a resistant starch if justification for the compound fulfilling criteria as a resistant starch were explicitly stated. Resistant starch to be administered at a dose of ≥10 g/day for ≥3 wk. Trials that included other interventions (e.g., drug use) were included if the effect of the resistant sarch alone could be isolated. Multiple intervention arms were eligible.	Resistant Starch type, placebo type, intervention and placebo dosage, duration of intervention
**Comparators**	Only studies with control group were included, The effect of the Resistant starch alone had to be able to be isolated.	Type and dose of comparator, compliance
Outcomes	Mean changes and SD in IL-6, CRP, hs-CRP and TNF-α	Outcomes measured, Evaluation methods and side effects.
Study design	Only randomized controlled trials, where it was possible to extract data on just the resistant starch compared with to placebo. We included both the parallel and crossover design	Design of the study, loss of the study, study quality

^a^ PICOS, participants, intervention, comparator, outcome, study type

For evaluation of the studies quality, we used from the Jadad Scale and the Downs and Black assessment tools [23, 24]. This checklist contain four domains including:: 1) randomization (mentioned as randomized, 1 point; mentioned appropriate randomization method, additional points), 2) blinding (mentioned as double blind, 1 point; mentioned appropriate blinding method, additional points) and 3) follow-up (the fate of all participants contains the number and the reason of participants dropouts, 1 point). Total score for this scale ranged 0 to five that score ≥ 3 indicate good quality and < 3 indicate poor quality [23]. The Downs and Black Scale consists of 27 questions relating to quality of reporting (10 questions), external validity (3 questions), internal validity (bias and confounding) (13 questions), and statistical power (1 question) [24].

### Data synthesis

We used from the effect size, as estimated by the mean difference to perform the fixed method meta-analysis. Weighted mean difference (WMD) with 95% CI was used for pooling data to determine effect sizes. For each measurement, a random effects meta-analysis was performed. I^2^ index was used for evaluation of heterogeneity. In the classification of I^2^ index, lower heterogeneity defined as a I^2^ < 30%, moderate if I^2^ = 30–75%, and high if I^2^ > 75% [25]. To find probable sources of between-study heterogeneity, subgroup analysis was done based on the study quality and intervention duration.

Funnel plots were used to visually inspect for the presence of publication bias. Moreover, we used from Begg’s rank correlation and Egger’s linear regression tests for evaluation of publication bias. All analyzes were performed using Stata software and results was regarded significant at *P* < 0.05.

## Results

**Fig. 1:**
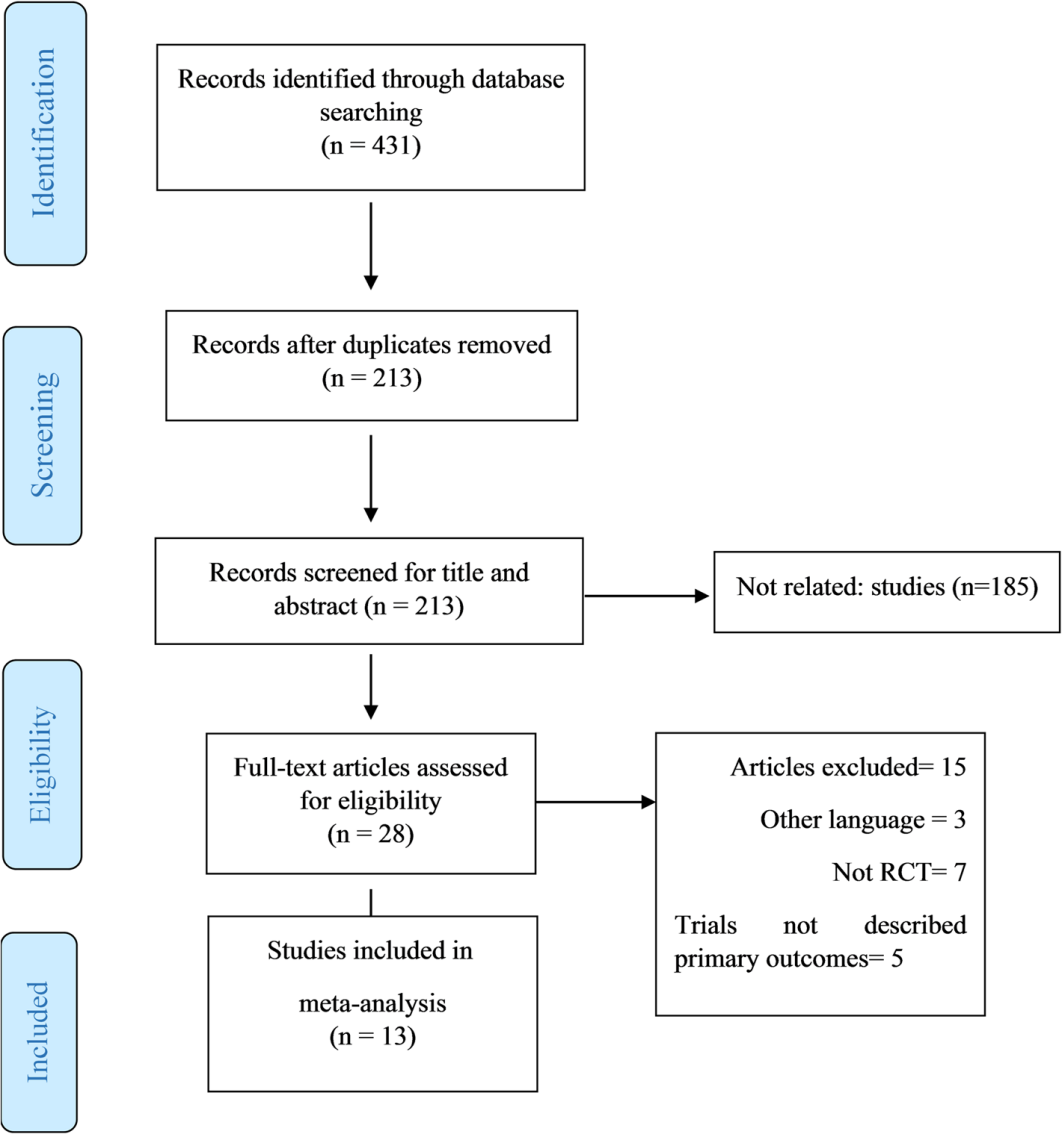
Flow diagram of literature search according to the PRISMA statement

Flow diagram of study selection is shown in Fig. 1. In total, 431 publications were found through the initial search; among them, 213 were entered in the second screening stage. Two researchers (MR and MV) independently evaluated the articles in the second screening stage and 185 studies that were not relevant were eliminated. Finally 13 studies with 14 effect sizes met the inclusion criteria and were included in the final analysis [17, 18, 20, 26–35].

### Study characteristics

The publications included in the meta-analysis are described in Table 2. Out of 13 articles that included in the final analysis, three study had cross-over [32, 33, 35] design and others had parallel design. Trials were conducted in Iran [18, 20, 26, 29, 31, 34], USA [17, 35], Canada [27], China [28], Denmark [33], France [32] and Brazil [30]. These studies were published between 2011 to 2019. Sample size of these studies ranged from 15 to 75 and intervention duration ranged from 4 to 14 weeks. In total, 672 participants (329 in the intervention group and 343 in the control group) included in the final analysis.

**Table 2:** Characteristics of included studies in the systematic-review^1^

First Author, Year (Ref)	Study design	Country	Age range	Gender	Participants (Intervention/control)	Intervention Type	Duration/week	Intervention Dose	Notes about subjects	Study quality	outcome
Aliasgharzadeh et al. [26]	RCT-parallel	Iran	30–65	F	30/25	resistant dextrin/maltodextrin	8	10	type 2 diabetes	G	IL-6, TNF-a, CRP
Peterson et al. [17]	RCT-parallel	United States	35–75	M/F	29/30	HAM-RS2/Amylopectin	12	45	prediabetes	F	TNF-a
Alfa et al. [27]	RCT-parallel	Canada	30–50	M/F	21/21	MSprebiotic/amioca	12	30	Healthy subjects	G	TNF-a, CRP
Karimi et al. [20]	RCT-parallel	Iran	30–65	F	28/28	Hi-maize 260/ maltodextrin	8	10	type 2 diabetes	G	CRP
Tayebi Khosroshahiet al [18].	RCT-parallel	Iran	≥18	M/F	23/21	HAM-RS2/waxy starch	8	20 g/d during first 4wk and 25 g/d during second 4 wk	patients on maintenance hemodialysis	G	CRP IL-6, TNF-a
Meng et al. [28]	parallel, open-label trial	China	18–80	M/F	34/36	high-RS,low-protein flour	12	17	Patients With Early Type 2 Diabetic Nephropathy	P	
Laffin et al. [29]	RCT-parallel	Iran		M/F	9/11	HAM-RS2/regular wheat flour	8	20 g/d during first 4wk and 25 g/d during second 4 wk	end-stage renal disease patients	G	IL-6, TNF-a
Esgalhado et al. [30]	Pilot RCT-parallel	Brazil	≥18	M/F	15/26	Hi-maize 260/manioc flour	4	16	hemodialysis patients	G	IL-6, CRP
Pourghassem Gargari et al. [31]	RCT-parallel	Iran	30–65	F	28/32	Hi-maize 260/maltodextrin	8	10	type 2 diabetes	G	IL-6, TNF-a, CRP
Lambert-Porcheron et al. [32]	randomized cross-over	France	20–65	M/F	20/20	high slowly digestible starch/low slowly digestible starch	6	10	healthy overweight subjects with metabolic risk	P	TNF-a, CRP
Schioldan et al. [33]	cross-over	Denmark	39–75	M/F	19/19	67 g dietary fiber(16 g arabinoxylan+ 21 g RS)/ 18 g dietary fiber (4 g arabinoxylan+ 3 g RS)	10	21	Metabolic syndrome	P	IL-6
Gholizadeh Shamasbi et al. [34]	RCT-parallel	Iran	18–45	F	31/31	resistant dextrin/ maltodextrin	12	20	polycystic ovarian syndrome	G	IL-6
Penn-Marshall et al. [35]	cross-over	USA	Mean: 36.6 ± 1.55	M/F	15/15	Hi-maize 260/no RS	8	12	Subjects at risk for type 2 DM.	P	CRP

*CRP* C - reactive protein, *IL-6* interleukin 6, *G* good quality, *F* fair quality, *P* poor quality, *RS* resistant starch

### Quality assessment

Findings from assessing the quality of RCTs are shown in Supplementary Table 1**.** According to the JADAD core, eight studies had high quality [17, 20, 26–28, 31, 32, 34] and five trials had low quality [18, 29, 30, 33, 35]. For a more accurate evaluation, we used the Downs and Black assessment tool as well, based on which seven trials had good quality (score > 19) [17, 20, 26, 27, 31, 32, 34], while six studies were deemed as low quality [18, 28–30, 33, 35], mostly due to lack of explanation of confounders and insufficient blinding.

### Findings from the meta-analysis of the effect of resistant starch on CRP levels

**Fig. 2:**
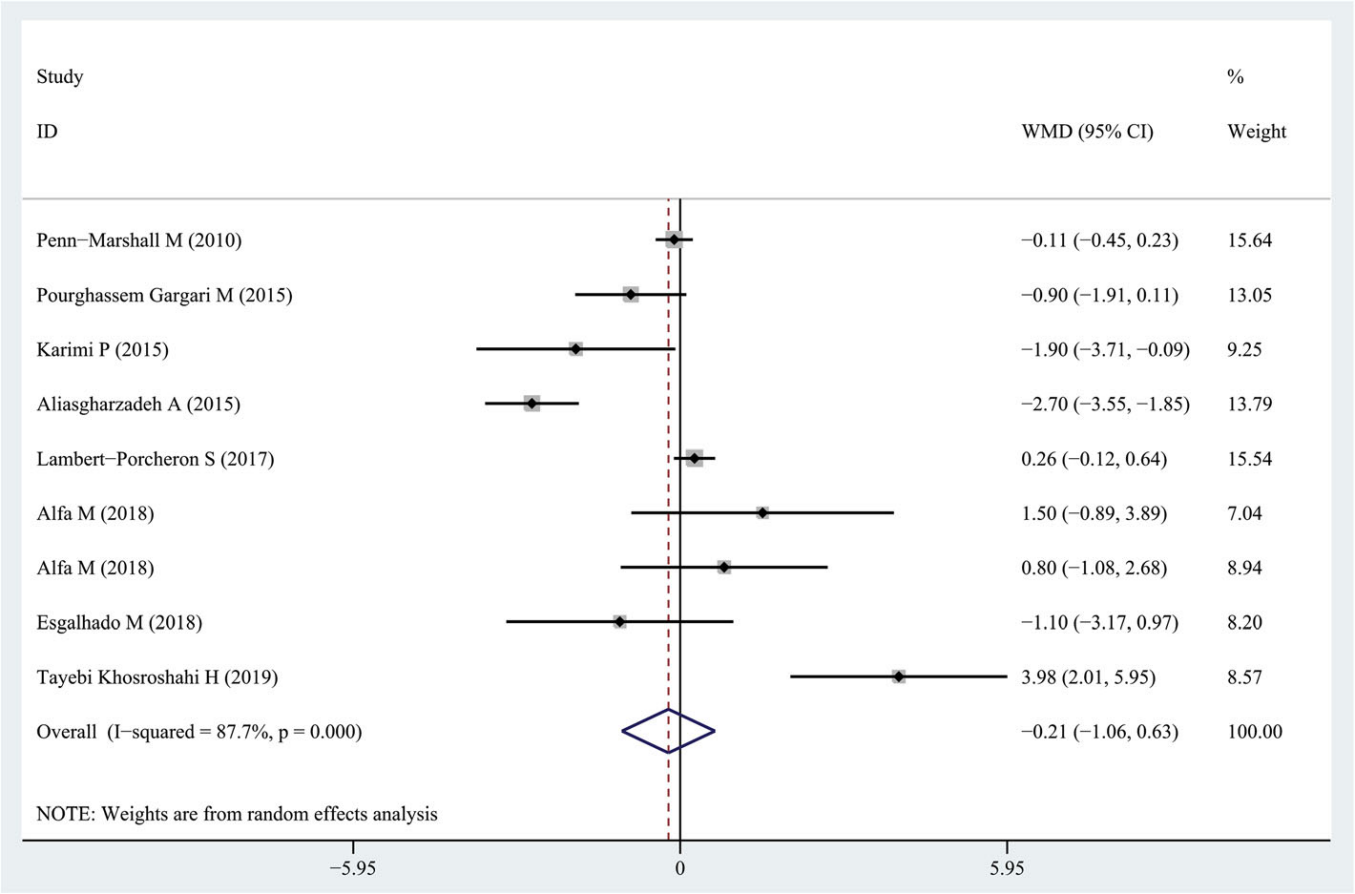
Forest plot summarizing the association between intake resistant starches on circulating CRP concentrations

In total, the effect of resistant starch supplementation on CRP levels was examined in 8 clinical trials with 9 effect size [18, 20, 26, 27, 30–32, 35], with a total 325 participants. Summarizing these effect sizes, we found that resistant starch consumption caused a non-significant reduction in the CRP concentration (weighed mean difference (WMD) = − 0.21 mg/L; 95% CI: − 1.06, 0.63 mg/L; *P* = 0.61), with a significant between-study heterogeneity (*I*^*2*^ = 87.7, *P* < 0.001) (Fig. 2). The subgroup analysis did not identify study quality (high or low) and intervention duration (> 12 weeks or lower) as sources of heterogeneity (Table 3). The sensitivity analysis did not provide any further information.

**Table 3:** Results of subgroup-analysis for effect of resistant starch on CRP and TNF-α and IL-6 levels

	No. of effect sizes	RR (95% CI)	P within^1^	I^2^ (%)	P between^2^
**Subgroup analyses for CRP and resistant starch**					
*Duration of follow up*					0.619
Less than 8 weeks	3	0.03 (−0.34, 0.4)	0.873	38.4	
8 weeks and more	6	− 0.21 (−1.06, 0.63)	0.998	89.7	
*Quality score*^3^					0.432
Scores≤median(19)	3	0.86 (− 1.63, 3.35)	0.498	88.3	
Scores>median(19)	6	−0.61 (− 1.93, 0.71)	0.364	89.3	
**Subgroup analyses for TNF-α and resistant starch**					
*Duration of follow up*					0.804
More than 8 weeks	4	−1.86 (−3.63, −0.09)	0.015	87.6	
8 weeks and less	4	−2.76 (−4.99, −0.54)	0.039	97	
*Quality score*^3^					0.562
Scores≤median(19)	2	−7.94 (−14.47, − 1.42)	0.017	76.8	
Scores>median(19)	6	−1.38 (−2.6, − 0.16)	0.026	94.8	
**Subgroup analyses for IL-6 and dairy products**					
*Duration of follow up*					0.001
More than 8 weeks	2	−0.48 (−1.61, 0.66)	0.163	62.8	
8 weeks and less	5	−1.40 (−2.22, − 0.58)	0.012	95.7	
*Quality score*^3^					0.001
Scores≤median(19)	4	−1.62 (−3.35, 0.12)	0.068	91.1	
Scores>median(19)	3	−0.97 (−1.81, −0.13)	0.024	99.5	

### Findings from the meta-analysis of the effect of resist ant starch on TNF-α levels

**Fig. 3:**
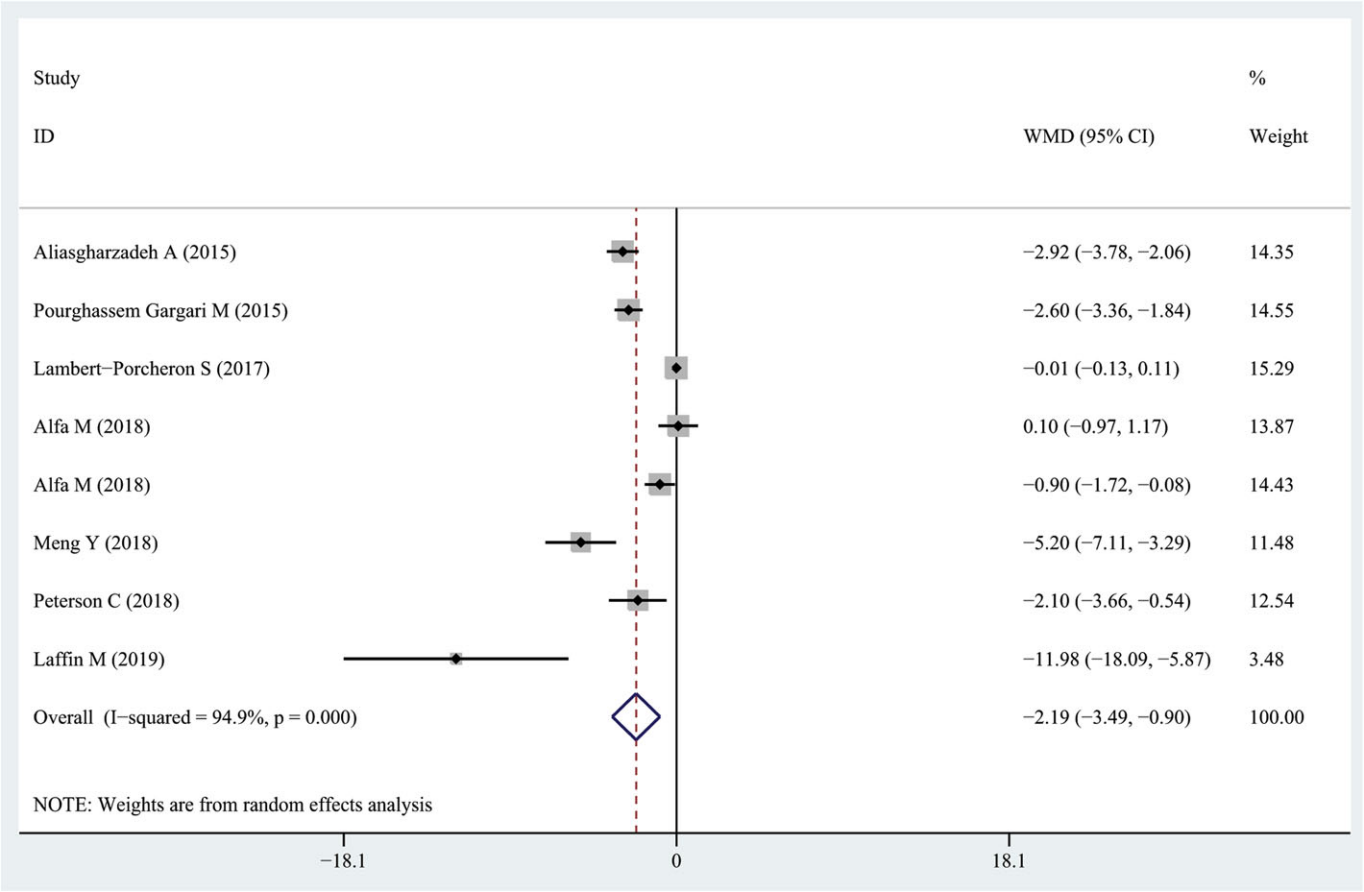
Forest plot summarizing the association between intake resistant starches on circulating TNF-α concentrations

Seven RCTs with eight effect sizes had reported the effect of resistant starch intake on TNF-α levels [17, 26–29, 31, 32]. Overall, we found that consumption of resistant starch could decrease serum TNF-α concentrations in comparison with the control group (WMD = − 2.19 pg/mL; 95% CI: − 3.49, − 0.9 pg/mL; *P* = 0.001) (Fig. 3). However, a significant between-study heterogeneity was found (*I*^*2*^ = 94.9, *P* < 0.001). Due to the high between-study heterogeneity, we stratified studies based on study quality (> 19 vs. ≤19) and duration of follow up (> 8 weeks vs. ≤8 weeks). The subgroup analysis did not provide additional information. Sensitivity analysis revealed that no individual study had a great effect on the overall results.

### Findings from the meta-analysis of the effect of resistant starch on IL-6 levels

**Fig. 4:**
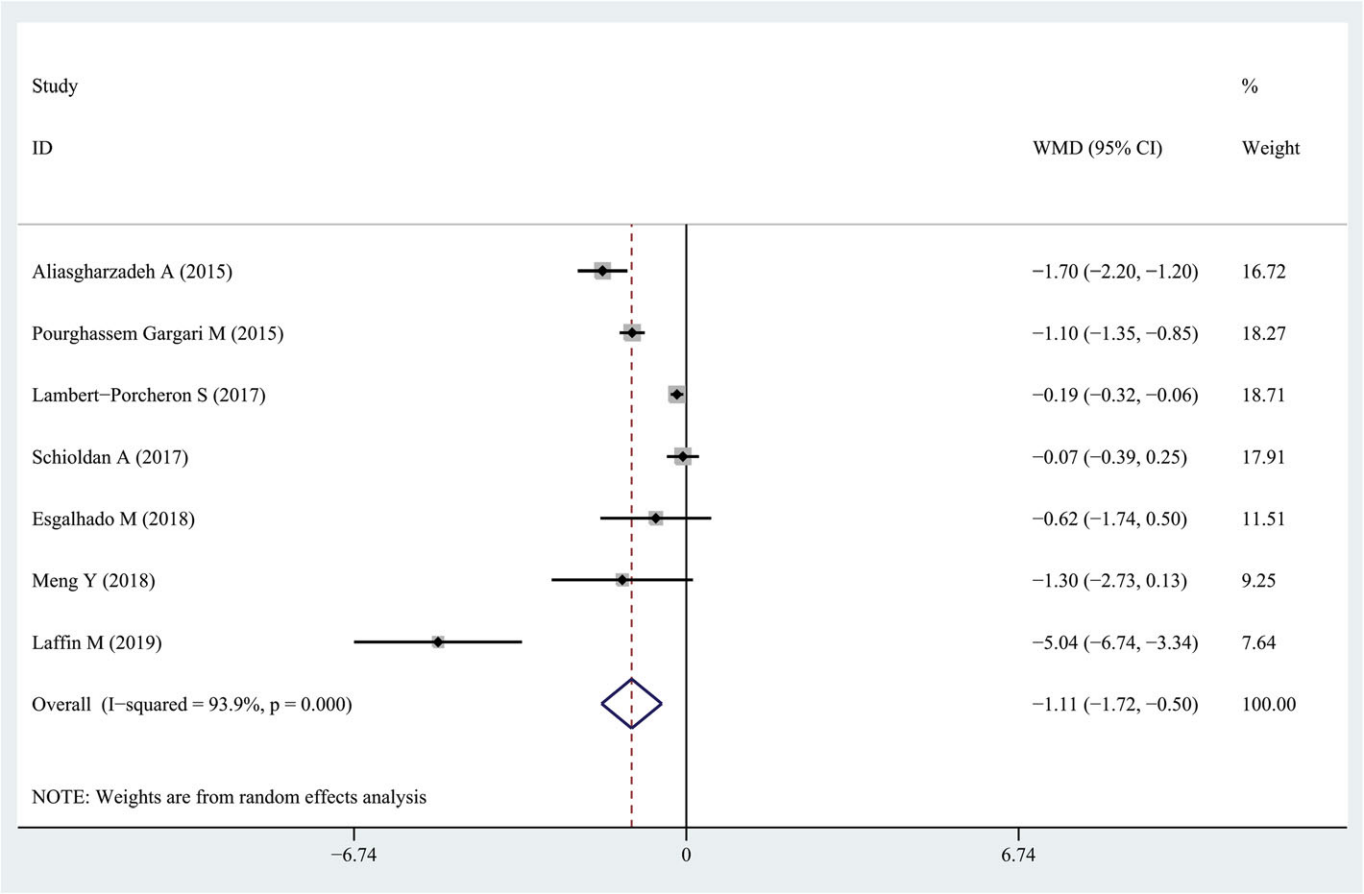
Forest plot summarizing the association between intake resistant starches on circulating IL-6 concentrations

Pooling effect sizes from seven studies [26, 28–33], the effect of resistant starch supplementation on serum IL-6 concentrations was significant (WMD = − 1.11 pg/mL; 95% CI: − 1.72, − 0.5 pg/mL; P = < 0.001) with a significant heterogeneity (*I*^*2*^ = 93.9, *P* < 0.001) (Fig. 4). Subgroup analysis based on study quality (> 19 vs. ≤19) and duration of follow up (> 8 weeks vs. ≤8 weeks) revealed a significant change in serum IL-6 concentrations in the high quality studies (WMD = − 0.97 pg/mL; 95% CI: − 1.81, − 0.13 pg/mL; *P* = 0.024) and that duration of follow up ≤8 weeks (WMD = − 1.40 pg/mL; 95% CI: − 2.22, − 0.58 pg/mL; *P* = 0.001). Sensitivity analysis revealed that no individual study had a great effect on the overall results.

### Publication bias

**Fig. 5:**
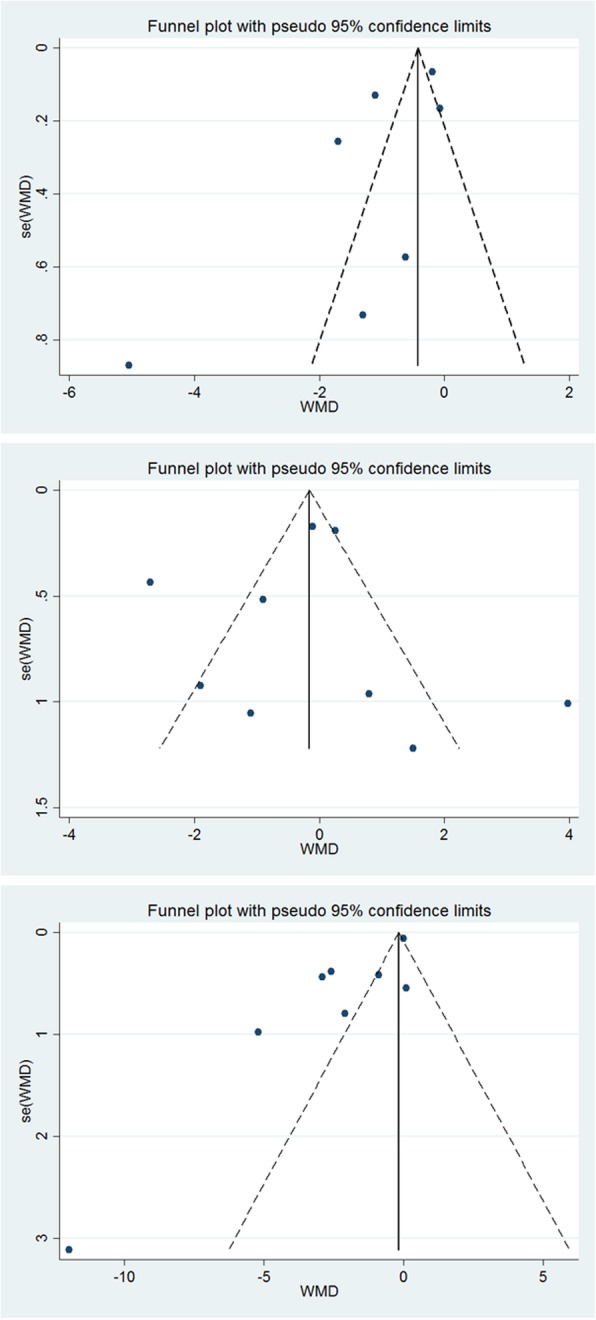
**a** Funnel plots detailing publication bias in the selected studies of the relation between intakes of resistant starches products and circulating IL-6. **b**. Funnel plots detailing publication bias in the selected studies of the relation between intakes of resistant starches products and circulating CRP. **c**. Funnel plots detailing publication bias in the selected studies of the relation between intakes of resistant starches products and circulating IL-6

Visual inspection of the funnel plots demonstrated no publication bias of the trials in investigating the effect of resistant starch intake on the IL-6 (Egger’s test *P* = 0.115; Begg’s test *P* = 0.24) (Fig. 5a) and CRP (Egger’s test *P* = 0.84; Begg’s test *P* = 0.91) (Fig. 5b) concentration. However, the funnel plot, Egger’s and Begg’s test showed a publication bias of the trials in investigating the effect of resistant starch supplementation on TNF-α concentration (Egger’s test *P* = 0.009; Begg’s test *P* = 0.013) (Fig. 5c).

## Discussion

As far as we know, this is the first systematic review and meta-analysis that surveyed the effect of resistant starch on circulating inflammatory biomarkers. The current study demonstrated that resistant starch consumption significantly reduced the levels of IL-6 and TNF-a, while it had no effect on CRP levels.

RS is a type of dietary fiber and can be fermented to SCFA (acetate, propionate, and butyrate) by intestinal bacteria. Butyrate is the main SCFA that is produced from the fermentation of RS and acts as an anti-inflammatory agent through interference in various inflammatory pathways [11–13]. The SCFA produced by RS fermentation are the main food source for anti-inflammatory regulatory T lymphocytes [31]. One of the proposed mechanism of reducing inflammation through SCFA, especially butyrate, is to inhibit NF-κB activation, which regulates inflammatory cytokines and chemokines [32]. Butyrate also controls the inflammation through increasing the expression of suppressor of cytokine signaling 3 (SOCS3) [33]. The aforementioned changes differentiate lymphocytes into Th2 rather than Th1 cells. Th2 decreased the production of inflammatory cytokines through Toll Like receptor 4-dependent signaling pathway by activating peroxisome proliferator-activated receptor gamma (PPAR- γ) [34]. Increased population of the symbiotic anti-inflammatory bacteria including Bifidobacterium and Faecalibacterium prausnitzii after inulin-type fructans supplementation could be another contributing mechanism [35].

Moreover, consuming RS through the weight loss, especially in obese and overweight people, can help to reduce inflammation. Excess body weight can increase the expression of inflammatory biomarkers such as TNF-a [36]. The exact mechanisms of weight reduction by RS remains unclear. However, some studies have reported that intake of RS increases serum concentration of leptin and other gut satiety hormones [37, 38]. Also, based on the previous studies, RS consumption could decrease metabolic endotoxaemia. Endotoxin levels usually higher in the patients with metabolic syndrome and other chronic disorders. Increased endotoxin levels (metabolic endotoxaemia) upregulated the expression of inflammatory cytokines [39, 40].

Some trials showed that RS can improve the levels of inflammatory markers [16–21], while in others this effect was not observed [22–27]. This paradox can be due to differences in starch type, duration of intervention, dose of intervention, health status of individuals, weight of individuals and etc.

The strengths of this study include identifying randomized trials with a rigorous search strategy and subgroup analysis of based study quality, and duration of intervention. Also, the results from the pooled effect size increased statistical power and are more convincing compared to a single study, considering the intra- and inter-individual variations as well as the small sample size of each eligible study.

Our studies has few limitations. Firstly, most of the studies that included in the final analysis had small sample size, which may bring a small study effect. Secondly, the samples were from various diseases and healthy individuals. Finally, the inter-study heterogeneity was high.

## Conclusion

In conclusion, the current study pooled results from 13 RCTs about the effects of RS consumption on inflammatory mediators. The results of our study showed that RS could have anti-inflammatory effects. Anyway, additional studies must be carried out that include well-designed protocols, and larger sample sizes to illustrate the beneficial effects of RS consumption on inflammation.

## Supplementary information

**Additional file 1 Supplementary Table 1-A.** Jadad Quality Assessment Scores^1.^**Supplementary Table 2.** Downs Quality Assessment Scores^1^.

## Data Availability

The datasets used and/or analyzed during the current study available from the corresponding author on reasonable request.

## Abbreviations

CRP: C-reactive protein
Hs-CRP: high-sensitivity C-reactive protein
IL-6: interleukin 6
NF-κB: Nuclear Factor Kappa B
RS: Resistant starch
SCFA: short chain fatty acids
TNF-α: tumor necrosis factor-α
WMD: Weighted Mean Difference
SOCS3: suppressor of cytokine signaling 3
PPAR- γ: proliferator-activated receptor gamma
