# Supplementary material for: Effects of resistant starch interventions on circulating inflammatory biomarkers: a systematic review and meta-analysis of randomized controlled trials
Source: Nutr J. 2020 Apr 15;19:33. doi: 10.1186/s12937-020-00548-6 (PMC7158011; doi:10.1186/s12937-020-00548-6)
Supplement: Supplementary file 1 — Additional file 1 Supplementary Table 1-A. Jadad Quality Assessment Scores1.Supplementary Table 2. Downs Quality Assessment Scores1. [file 12937_2020_548_MOESM1_ESM.docx]

**Supplementary Material**

Mahsa Vahdat; Seyed Ahmad Hosseini; Golsa Khalatbari Mohseni, Javad Heshmati, Mehran Rahimlou

**Supplementary Table 1-A.** Jadad Quality Assessment Scores^1^.

| **First author of the study**, Year (Ref.) | Blinding | Randomization | Withdrawals | score |
| --- | --- | --- | --- | --- |
| Marshall, 2008 (36) | 0 | 1 | 1 | 2 |
| Karimi, 2015 (20) | 0 | 2 | 1 | 3 |
| Aliasgharzadeh 2015 (26) | 2 | 2 | 1 | 5 |
| Gargari 2015 (32) | 2 | 2 | 1 | 5 |
| Lambert, 2017 (33) | 1 | 2 | 1 | 4 |
| Schioldan 2017 (34) | 0 | 1 | 0 | 1 |
| Peterson, 2018(17) | 1 | 2 | 1 | 4 |
| Alfa, 2018 (27) | 2 | 2 | 1 | 5 |
| Meng, 2018 (29) | 0 | 2 | 1 | 3 |
| Esgalhado, 2018 (31) | 0 | 1 | 1 | 2 |
| Gholizadeh 2018(35) | 1 | 2 | 1 | 4 |
| Tayebi, 2018(18) | 0 | 1 | 1 | 2 |
| Laffin, 2019 (30) | 0 | 1 | 1 | 2 |

^1^A.R. Jadad, R.A. Moore, D. Carroll, C. Jenkinson, D.J.M. Reynolds, D.J. Gavaghan, et al. Assessing the quality of reports of randomized clinical trials: is blinding necessary?Control Clin Trials, 17 (1996), pp. 1-12

**Supplementary Table 2.** Downs Quality Assessment Scores^1^

| **Assessment Question** |  |  | |  | |  |  |  |  |  |  |  |  |  |  |  |
| --- | --- | --- | --- | --- | --- | --- | --- | --- | --- | --- | --- | --- | --- | --- | --- | --- |
|  | Marshall 2010 (33) | Karimi  2015  (19) | Gargari  2015 | | Aliasgharzadeh  2015 | | | Lambert 2017  (18) | Schioldan  2017 | Peterson 2018  (32) | Alpha  2018  (29) | Meng  2018  (34) | Esgalhado 2018  (31) | Gholizadeh 2018  (36) | Tayebi  2018 | Laffin 2019  (30) |
| 1. Hypothesis clearly described? | 1 | 1 | 1 | | 1 | | | 1 | 1 | 1 | 1 | 1 | 1 | 1 | 1 | 1 |
| 2. Main outcomes listed in the introduction methods? | 1 | 1 | 1 | | 1 | | | 1 | 1 | 1 | 1 | 1 | 1 | 1 | 1 | 1 |
| 3. Included patient characteristics clearly described? | 1 | 1 | 1 | | 1 | | | 1 | 0 | 1 | 1 | 0 | 1 | 1 | 0 | 0 |
| 4. Interventions of interest clearly described? | 1 | 1 | 1 | | 1 | | | 1 | 1 | 1 | 1 | 1 | 1 | 1 | 1 | 1 |
| 5. Principle confounders clearly described? | 0 | 0 | 1 | | 1 | | | 0 | 0 | 0 | 1 | 0 | 0 | 1 | 0 | 0 |
| 6. Main findings clearly described? | 1 | 1 | 1 | | 1 | | | 1 | 1 | 1 | 1 | 1 | 1 | 1 | 1 | 1 |
| 7. Provide estimates of random variability? | 1 | 1 | 1 | | 1 | | | 1 | 1 | 1 | 1 | 1 | 1 | 1 | 0 | 1 |
| 8. Adverse events of the intervention reported? | 0 | 1 | 0 | | 1 | | | 0 | 0 | 1 | 0 | 1 | 0 | 0 | 0 | 0 |
| 9. Patient loss to follow-up been described | 1 | 1 | 1 | | 1 | | | 1 | 0 | 1 | 1 | 1 | 1 | 1 | 0 | 0 |
| 10. Actual probability values reported? | 0 | 0 | 0 | | 0 | | | 1 | 1 | 1 | 1 | 1 | 1 | 1 | 0 | 1 |
| 11. Subjects representative of entire population? | 1 | 1 | 1 | | 1 | | | 1 | 1 | 1 | 1 | 1 | 1 | 1 | 1 | 1 |
| 12. Subjects representative of entire population recruited? | 0 | 1 | 1 | | 1 | | | 1 | 1 | 1 | 1 | 1 | 1 | 1 | 1 | 1 |
| 13. Facilities representative of treatment majority of patients receive? | 0 | 1 | 1 | | 1 | | | 1 | 1 | 1 | 1 | 1 | 0 | 1 | 1 | 0 |
| 14. Attempt to blind study subjects? | 0 | 1 | 1 | | 1 | | | 0 | 0 | 1 | 1 | 0 | 1 | 1 | 1 | 1 |
| 15. Attempt to blind researchers? | 0 | 0 | 1 | | 1 | | | 0 | 0 | 1 | 1 | 0 | 1 | 1 | 1 | 1 |
| 16. Data dredging reported? | 1 | 1 | 1 | | 0 | | | 1 | 0 | 1 | 0 | 0 | 1 | 0 | 0 | 1 |
| 17. Adjustment for length of follow-up? | 0 | 0 | 0 | | 0 | | | 0 | 0 | 0 | 0 | 0 | 0 | 0 | 0 | 0 |
| 18. Statistical test appropriate? | 1 | 1 | 1 | | 1 | | | 1 | 1 | 1 | 1 | 1 | 1 | 1 | 1 | 1 |
| 19. Compliance with interventions reliable? | 0 | 0 | 0 | | 0 | | | 1 | 0 | 1 | 1 | 1 | 0 | 1 | 0 | 0 |
| 20. Main outcomes measures used accurate? | 1 | 1 | 1 | | 1 | | | 1 | 1 | 1 | 1 | 1 | 1 | 1 | 1 | 1 |
| 21. Different intervention groups recruited from same population? | 1 | 1 | 1 | | 1 | | | 1 | 1 | 1 | 1 | 1 | 1 | 1 | 1 | 1 |
| 22. Different intervention groups recruited over same period of time? | 1 | 1 | 1 | | 1 | | | 1 | 1 | 1 | 1 | 1 | 1 | 1 | 1 | 1 |
| 23. Subjects randomized? | 1 | 1 | 1 | | 1 | | | 1 | 1 | 1 | 1 | 1 | 1 | 1 | 1 | 1 |
| 24. Randomization concealed from patients and healthcare staff? | 0 | 0 | 1 | | 1 | | | 0 | 0 | 0 | 0 | 0 | 0 | 0 | 0 | 0 |
| 25. Adjustment for confounding in the analysis? | 0 | 1 | 1 | | 1 | | | 1 | 0 | 1 | 0 | 1 | 0 | 0 | 0 | 1 |
| 26. Patient loss to follow-up been taken into account in analysis? | 1 | 1 | 1 | | 1 | | | 1 | 0 | 1 | 1 | 1 | 1 | 1 | 1 | 1 |
| 27. Power calculation reported? | 0 | 1 | 1 | | 1 | | | 1 | 0 | 1 | 1 | 0 | 0 | 0 | 0 | 0 |
| **Total Score** | 15 | 21 | 23 | | 23 | | | 21 | 14 | 22 | 22 | 19 | 19 | 21 | 15 | 18 |

^1^S. H. Downs, N. Black. The feasibility of creating a checklist for the assessment of the methodological quality both of randomised and non-randomised studies of health care
